# Digital Microlearning for Training and Competency Development of Older Adult Care Personnel: Mixed Methods Intervention Study to Assess Needs, Effectiveness, and Areas of Application

**DOI:** 10.2196/45177

**Published:** 2023-12-04

**Authors:** Matt X Richardson, Osman Aytar, Katarzyna Hess-Wiktor, Sarah Wamala-Andersson

**Affiliations:** 1 Department of Health and Welfare Technology School of Health, Care and Social Welfare Mälardalen University Västerås Sweden; 2 Department of Social Work School of Health, Care and Social Welfare Mälardalen University Västerås Sweden; 3 Minnity AB Stockholm Sweden

**Keywords:** digital microlearning, elderly care, older adult care, competency development, implementation research, dementia, COVID-19

## Abstract

**Background:**

Older adult care organizations face challenges today due to high personnel turnover and pandemic-related obstacles in conducting training and competence development programs in a time-sensitive and fit-for-purpose manner. Digital microlearning is a method that attempts to meet these challenges by more quickly adapting to the educational needs of organizations and individual employees in terms of time, place, urgency, and retention capacity more than the traditional competency development methods.

**Objective:**

This study aimed to determine if and how an app-based digital microlearning intervention can meet older adult care organizations’ personnel competency development needs in terms of knowledge retention and work performance.

**Methods:**

This study assessed the use of a digital microlearning app, which was at the testing stage in the design thinking model among managerial (n=4) and operational (n=22) employees within 3 older adult care organizations. The app was used to conduct predetermined competency development courses for the staff. Baseline measurements included participants’ previous training and competency development methods and participation, as well as perceived needs in terms of time, design, and channel. They then were introduced to and used a digital microlearning app to conduct 2 courses on one or more digital devices, schedules, and locations of their own choice during a period of ~1 month. The digital app and course content, perceived knowledge retention, and work performance and satisfaction were individually assessed via survey upon completion. The survey was complemented with 4 semistructured focus group interviews, which allowed participants (in total 16 individuals: 6 managerial-administrative employees and 10 operational employees) to describe their experiences with the app and its potential usefulness within their organizations.

**Results:**

The proposed advantages of the digital microlearning app were largely confirmed by the participants’ perceptions, particularly regarding the ease of use and accessibility, and efficiency and timeliness of knowledge delivery. Assessments were more positive among younger or less experienced employees with more diverse backgrounds. Participants expressed a positive inclination toward using the app, and suggestions provided regarding its potential development and broader use suggested a positive view of digitalization in general.

**Conclusions:**

Our results show that app-based digital microlearning appears to be an appropriate new method for providing personnel competency development within the older adult care setting. Its implementation in a larger sample can potentially provide more detailed insights regarding its intended effects.

## Introduction

Adequate training and competence development among personnel in older adult care organizations are not only vital for their health, well-being, safety, and self-confidence but also for the outcomes of care receivers. Such development minimizes ill-health and related leave of absences and enhances staff continuity [1]. Older adult care in Sweden is faced with several concurrent challenges to this kind of training and development. These include high staff turnover, staff with shorter education, inadequate skills, and limited Swedish language proficiency. For this group, standard training and competence development programs are less prioritized due to heavy workload, more difficult to conduct, and when conducted are less likely to give expected benefits [2].

Regulations regarding infectious disease control as a response to the COVID-19 pandemic also created a dramatic change in work methods, affecting both skills and competency requirements. Fit-for-purpose training and development initiatives need to be provided in a safe and effective manner despite changes in work routines and limitations on physical gathering and contact. The pandemic also complicated the “feedback loop” between employees and management regarding knowledge and competency gaps, making them more difficult to identify or remedy in time and scope. These developments made many work environments less adaptable in considering the personnel’s health, safety, and development and led to adverse work–related outcomes and increased absence due to illness [3].

Digital training and competency development tools have the potential to ameliorate some of these challenges. One such tool, digital microlearning, is a less formal competency development tool that aims to fill knowledge gaps identified by the staff themselves through self-assessment, known as personalized learning. To achieve this, digital microlearning provides brief learning modules (seconds to minutes to conduct) on demand via digital means (eg, computers, mobile telephones, or tablets) that are highly specific to the individual’s learning context and environment. The method often includes an assessment that is provided in close proximity to the learning modules in terms of timeframe and delivery channel. Digital microlearning aims to increase flexibility in organizations’ competency development compared to standard and more formal methods, which are often delivered broadly through written course literature and lectures with a longer time lag between current needs and delivery. Organizations undergoing change may benefit from shorter, more focused, and directed training efforts that can be delivered on short notice to specific roles and individuals [4,5]. A recent review of microlearning in health professions training concluded that, as a competence development strategy, it contributed to positive effects in actual knowledge retention as well as self-confidence in work performance compared to standard training methods [6]. This may be due to a reduction in the information load associated with the broader scope and time allotment of standard formal training [7]. Furthermore, many digital microlearning tools reduce the administrative burden on managers in documenting and following employees’ learning progress toward set goals.

The use of digital microlearning among health and social care settings and professionals is not well documented in the research literature, although its use in some thematic materials relevant to these professions, such as dealing with violent behavior and mental health issues, have been attempted [6].

This study focused on health and social care organizations providing home and institutional care to older persons, to answer the following research questions:

1. What are the competency development needs of employees and managers in older adult care organizations in terms of subject matter, time, method of provision, and channels?
2. Can digital microlearning meet the identified needs of employees and managers in older adult care organizations), and if so, how can the efficiency of delivery and effectiveness of retention and app be improved?
3. Are professionals’ own assessments of confidence in their knowledge and work performance affected by digital microlearning, and if so, how?

**Figure 1:**
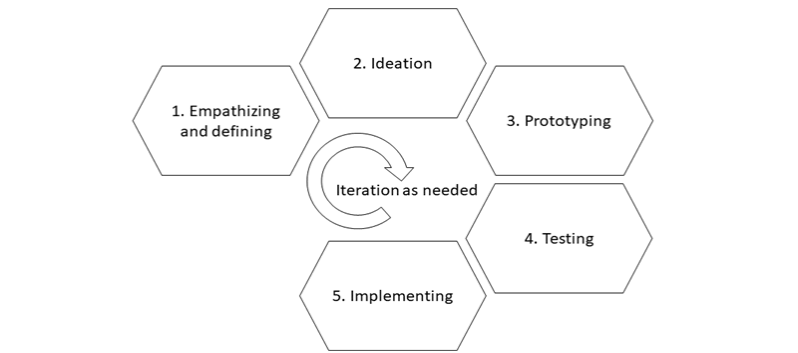
The stages of the Design Thinking Model [8]. The digital microlearning app in this study was in stage 4 at the time of the study and had undergone previous iterative rounds of the other stages.

To answer research questions 2 and 3, the study used a digital microlearning app (The Minnity Learning Platform) that was in the later iterative stages of test mode according to the design thinking model [8]. This study evaluated the effects of a digital microlearning app at the testing stage (stage 4) in the design thinking model (Figure 1). Stages 1-3 in the model had already been conducted in other populations, although some steps in these stages were repeated in this study. A codevelopment process for the app was used involving the developer, municipal caregiving organizations, and the academic research sector.

## Methods

### Recruitment and Participants

Recruitment of participants for the study was conducted between January and May 2021. Targeted offers to participate in the study were sent to 6 health and social care organizations providing home- and institution-based older adult care. The offers specified that a small reimbursement (~US $1200) would be given for their time. Three organizations volunteered to participate in the study, each located in different Swedish municipalities with comparatively diverse demographic characteristics. Managerial or administrative employees (n=4) and operational caregiving employees (n=22) without previous experience with microlearning methods were internally recruited within the organizations to voluntarily participate in the study (26 in total). Participants then received both verbal and written information about the aims of the study, their expected contributions, potential benefits and risks, and how data and personal information gathered during the study would be used in line with recommendations on good research practice from the Swedish Research Council [9] and the EU General Data Protection Regulation. All participating organizations and employees then provided informed consent verbally and in writing to participate in the study. Background data regarding the organizations and the employee participants were collected from the respective organizations’ official administrational data. The three participating organizations and the recruited participants from these organizations are described in Table 1.

**Table 1:** Characteristics of the participating organizations and employees.

	Location	Turnover (2020); number of employees	Care receivers, n	Study participants
Organization 1: unit within municipal caregiving company	Small municipality (<30,000 inhabitants)	~US $40 million; 20-25 employees	50-75; with addition of several hundred home emergency alarm users	Nine nursing assistants and 1 manager; average age 49 years (between 35 and 60 years); average employment experience 13 years (between 0 and 35 years)
Organization 2: unit within the caregiving foundation	Large municipality (~1,000,000 inhabitants)	~US $6.5 million; 60-65 permanent contract employees	65-70	Four nursing assistants, 2 operational team managers, and 2 administrative managers; average age 51 years (between 35 and 65 years); average employment experience 6 years (between 0 and 15)
Organization 3: municipal caregiving unit	Small municipality (<35,000 inhabitants)	US $2.5 million; 30-35 permanent contract employees	30-35	Six nursing assistants, 1 operational team manager, and 1 administrative manager; average age 48 years (35-65 years); average employment experience 13 years (0-25)

### Intervention

#### Overview

The study intervention was conducted between April and November 2021. It consisted of three stages: (1) characterization and assessment of previous competence development and identification of current or future needs, (2) implementation and evaluation of a market-ready digital microlearning app and courses, and (3) assessment of the app’s potential usefulness in meeting future competence development needs.

#### Characterization and Assessment of Previous Competence Development and Identification of Current or Future Needs

A web-based survey of all participants was then conducted to obtain individual responses regarding competence development initiatives conducted during the previous 2 years of employment, including themes or subject matter, time allotted to, channels and evaluation methods used, and experiences and satisfaction with such initiatives. For the experiences and satisfaction dimensions, the survey posed statements that the respondent should then choose an appropriate response to from a 5-point Likert scale, with the response alternatives “completely agree,” “mostly agree,” “both agree and disagree,” “mostly disagree,” and “completely disagree” (see Multimedia Appendix 1). The survey also addressed desired support, needs, and pandemic-related aspects regarding current and future competence development. All participants were given approximately 1 month to complete the survey.

#### Implementation and Evaluation of the Digital Microlearning App and Courses

Participants were then introduced to The Minnity Learning Platform digital microlearning app that was to be implemented as part of the study. The internet-based app could be used on mobile (smartphone and tablet) or computer platforms. All participants received 30 minutes of instructive group training on how to use the app, as well as unlimited access to web-based manuals and support afterward. Managers received additional training on how to monitor employees’ progress through the microlearning modules via the administrative view in the app.

**Figure 2:**
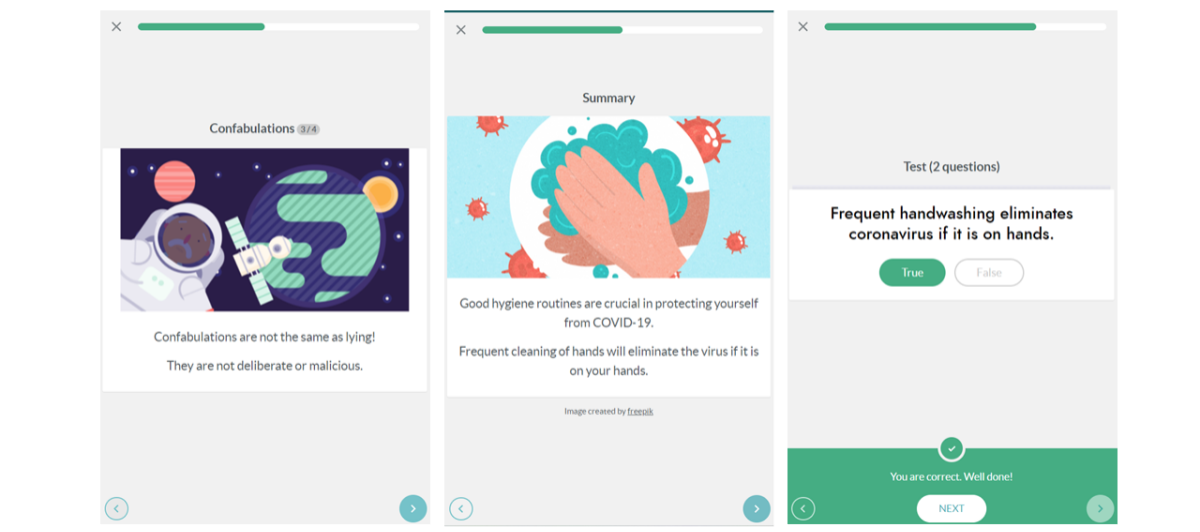
Screenshots from the mobile version of the digital microlearning app. From left to right: the dementia course, the hygiene course, and the self-assessment test for the hygiene course.

Two full microlearning courses were conducted via the app: (1) COVID-19 and hygiene when providing home care and (2) care approaches for people with dementia. (A trial of the COVID-19 module is available publicly [10] in English, French, and Swedish languages.) The courses consisted of several small modules, each of which was expected to take 2-3 minutes to complete, with a repeatable self-assessment test to be completed at the end of each module (Figure 2). The entire course therefore was expected to take approximately 15-20 minutes to complete but could be started and stopped after each module, and modules could be repeated as desired. Participants were given 1 month to complete the modules, during which time they could freely choose to conduct the modules as they wished. The progress of individuals through the modules could be seen by themselves as well as by the manager involved in the project from their organization.

Upon completing the course, individuals were then directed to a short (approximately 3 minutes) web-based survey conducted via a link from the app to evaluate their perceptions about the course content, its usefulness and applicability, and different user experience dimensions regarding the digital microlearning app itself (Multimedia Appendix 2). The surveys posed the same statements and used the same 5-point Likert scale as in the initial survey (characterization of previous competence development and identification of current or future needs section).

#### Assessment of the App’s Potential in Meeting Future Competence Development Needs

After all participants in an organization had completed the modules, 2 semistructured group interviews were conducted with the managerial-administrative participants and the operational participants, respectively. The approximately 1-hour interviews were conducted either physically or via web-based meeting as chosen by the participant organization (which had different restrictions on receiving external visitors during the COVID-19 pandemic), recorded, and transcribed. The interviews initially focused on three main themes: (1) the participants’ conduct of the digital microlearning courses, such as time, place, amount, and strategy, and their discussions with colleagues regarding the courses’ content; (2) their own assessment of the app’s and courses contents’ effects on their comprehension and retention of the courses’ content, as well as their confidence in and ability to apply the content material in their daily work; and (3) their own assessment of if and how the app could be used in the future within their organizations, how the current course content could be developed, and what other courses that would be useful to conduct. The interviewers assisted participants in identifying subthemes and additional themes during the interviews using the constant-comparison method [11]. Participants spoke freely both individually and among themselves during the interview. The interview guide used can be found in Multimedia Appendix 3.

### Statistical Analysis

For quantitative data, descriptive univariate analysis was applied across organizations, and results were presented as sums or averages (with range or SD where applicable). Within-groups and between-groups analyses were conducted for the organization (3 participating) and role within the organization (2 participating) variables.

Transcribed qualitative interview data were descriptively coded by 2 researchers (first level) to formulate a primary list of themes and associated citations, followed by second-level coding to expand or amalgamate themes. The themes and their content were summarized and designated as facilitating, hindering, or neutral by 2 researchers independently conducting the analysis.

### Ethical Considerations

Head administrators of the participating organizations, as well as the individual employees recruited within them, signed written informed consent forms to participate in the study after receiving oral and written information about the aims of the study, their expected contributions, potential benefits, and risks, and how data and personal information gathered during the study would be used. A nonconditional reimbursement of ~US $1200 was offered to organizations at the time of recruitment. Individual participants’ data were coded after data collection and remained anonymous to both researchers and others from that point onward. Participation was voluntary and could be ended at any point without explanation, at either the individual or the organizational level. Ethics approval by the Swedish Ethics Review Board was not sought as it was deemed, after following internal Mälardalen University process, that the study did not meet the requirements for mandatory national ethics review. The study did follow recommendations on good research practice from the Swedish Research Council [11] as well as adhering to the EU General Data Protection Regulation.

## Results

### Overview

Organizations 2 and 3 (n=16) completed all 3 stages of the study. Organization 1 (n=10) completed the first 2 stages of the study but not the third stage due to logistical difficulties (both directly and indirectly influenced by the ongoing pandemic) in participating in the interview.

### Characterization and Assessment of Previous Competence Development and Identification of Current or Future Needs (N=26)

**Figure 3:**
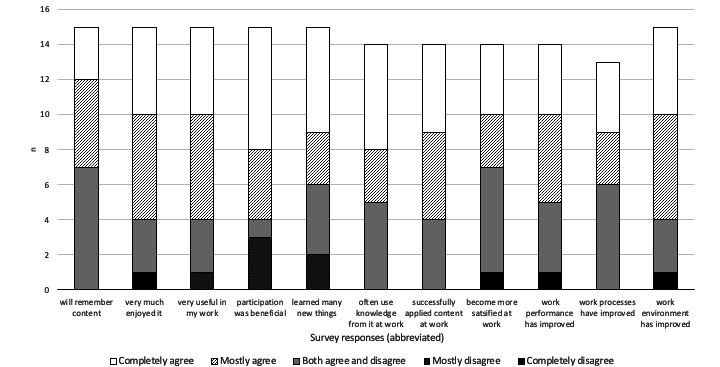
Participants’ (N=26) assessment of previous competency development and educational initiatives conducted within their respective organizations. The statements under the bars that participants stated their level of agreement about are shortened for space purposes; the full statements can be found in Multimedia Appendix 1.

For all organizations, 15 employees confirmed that they had participated in educational or competence development initiatives within the organization during the 2 years prior to the study. Of those confirmed, 6 had participated for 1-2 days per year, 3 had participated for 3-5 days per year, 1 had participated for 6-10 days, 3 had participated for 11-20 days per year, and 1 had participated for more than 20 days per year. There was no difference between organizations for these measures, although managerial and administrative employees on average participated in more initiatives (mean 12.5, SD 9.6 days) than operational employees (mean 1.6, SD 4.2 days). Web-based educational modules consisting of mixed methods (video and text lectures, quizzes, and assignments) were the most common form of competence development received (5 participants), followed by lectures (digital or in person; 4 participants). Workshops, internships or mentoring, and structured reading, study circles, written assignments, or other methods were also used. For conducting educational and competence development initiatives, the workplace was the most common site (65.2% of all initiatives), while educational institutions (8.7%), own residence, or other sites (13% each) were less used. Educators outside of the organization were most used for conducting the initiatives (35.7% of all initiatives), followed by web-based content (25%), internal educators (21.4%), paper literature (14.3%), and other resources (3.5%). The individual assessments of previously conducted education or competence development initiatives are shown in Figure 3.

### Evaluation of the Digital Microlearning Courses and App (N=26)

**Figure 4:**
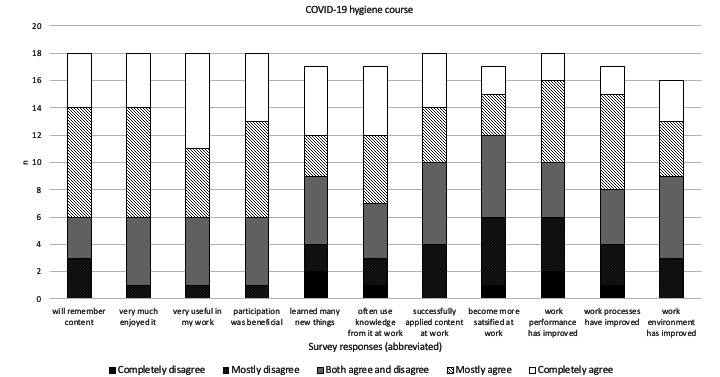
Participants’ (N=26) assessment of the COVID-19 hygiene digital microlearning course. The statements under the bars that participants stated their level of agreement about are shortened for space purposes; the full statements can be found in Multimedia Appendix 1.

**Figure 5:**
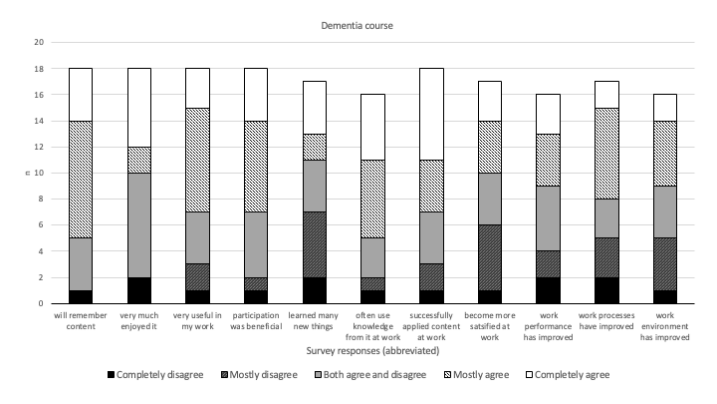
Participants’ (N=26) assessment of the dementia care digital microlearning course. The statements under the bars that participants stated their level of agreement about are shortened for space purposes; the full statements can be found in Multimedia Appendix 1.

The survey assessments of the digital microlearning courses showed that a majority of participants were entirely or mostly in agreement regarding the content in both the COVID-19 and dementia courses. The participants found the courses to be valuable and beneficial, with a high probability of retaining the acquired knowledge. They were able to apply this knowledge to enhance their work performance and to improve their work environment and workflows. Additionally, they expressed satisfaction and enjoyment in attending the course (Figures 4 and 5). However, the majority mostly disagreed or disagreed regarding satisfaction with work following attending the COVID-19 module, and new knowledge gained from the dementia module. No differences were found between operational and managerial-administrative participants’ responses regarding the courses.

**Figure 6:**
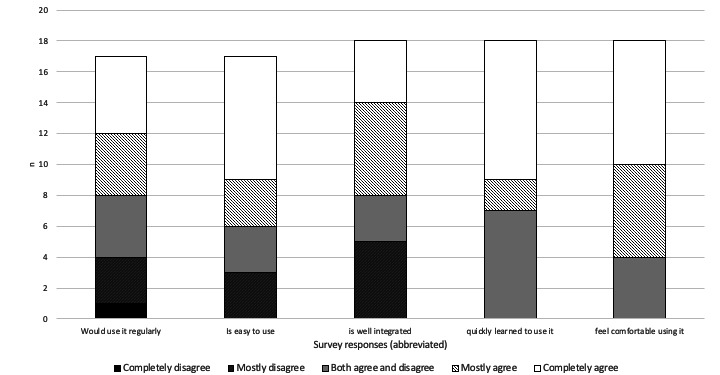
Participants’ (N=16) assessment of the digital microlearning app. The statements under the bars that participants stated their level of agreement about are shortened for space purposes; the full statements can be found in Multimedia Appendix 2.

A majority of the participants were also entirely or mostly in agreement that the digital microlearning app was simple to use and well integrated, and that they could quickly learn to use it, felt comfortable in using it, and wanted to use it regularly (Figure 6). There were no differences between the operational and managerial-administrative participants’ responses regarding the app.

### Assessment of the Digital Microlearning App’s Potential in Meeting Future Competence Development Needs (n=16)

Several future course topics were suggested as appropriate for the digital microlearning app in comparison to traditional planned educational initiatives, due to its more time-sensitive and on-demand nature, including:

- Protective and restrictive interventions for aggressive or violent care receivers, as well as management and prevention of other behavioral problems such as problematic anxiety and restlessness. These were seen as more acute in time and expedient access to knowledge would be particularly useful delivered through the app.
- Mealtime layout planning and related cultural knowledge in holiday meal settings. These events were often time specific and in some cases needed adjustment on a very short time scale, and the knowledge level of employees with non-Swedish ethnic and geographic backgrounds on this topic required additional support.
- Digital signing and transfer of responsibilities regarding, for example, care provision or medication, as well as documentation of, for example, adverse events or deviating procedures. These have strict local, regional, and national regulations that newer employees felt a need for support in adhering to.
- Purely methods-related knowledge, such as wound and sore management and how to apply insulin or eye drops in various care receiving groups.
- Areas of knowledge that changed quickly due to, for example, regulation development, societal or organizational changes, or research-based findings. The changing knowledge status during the COVID-19 pandemic was according to some a particularly relevant example of how needs could be more easily solved in a digital microlearning setting.

A key advantage of having these topics provided via a digital microlearning app was that several employees felt that knowledge retention would be greater with the ability to repeat the same educational modules. Previously offered educational initiatives were often single sessions and significant amounts of the material were quickly forgotten according to some. For example, 1 nurse summarized, “(The course material) is fresh in your mind while you’re there, but the forgetfulness sets in fast.” Another stated that with the application, “…you can have the material close to you all the time… and use it to quickly freshen up your memory.”

Other potential topics were seen as appropriate for the digital microlearning app, but in tandem or complementary to previously used planned educational initiatives as they could not provide the in-depth material, discussion of it with colleagues and instructors, or collegial guidance that the previously used methods enabled. As 1 nursing assistant stated:

Some education needs to be about ethics and dilemmas… and then it’s better to be in the same place with others and have a forum for discussion.

Examples of such topics included the following:

- Support to relatives and informal caregivers when the professional caregivers were not available or involved, regarding, for example, the management or transfer of certain care responsibilities requiring a higher level of knowledge or proficiency. The app was seen as useful in providing a similarly accessible and summary format to informal caregivers “on the spot.”
- End-of-life and palliative care routines, where certain recurring situations or events required expedient access to standard knowledge. The ethical and personal reflection regarding such situations was, however, not seen as appropriate for the app and required other educational approaches.

### Other User Groups’ Interaction With the Digital Microlearning App

Study participants also commented that the app would be useful for other user groups that had a stake in their own work activities, specifically managers within the organization, informal caregivers or relatives, and other relevant professional groups such as doctors and nurses within primary or secondary care, physiotherapists, and work therapists.

Managerial participants had access to a function that allowed them to see operational participants’ time and level of completion of the modules, and this function was considered useful so that reminders could be provided. This function was used during the study to remind operational participants who had not completed the courses that they were nearing the end of the designated period within the study.

For informal caregivers and relatives, a similar supportive function in obtaining new knowledge as for professional caregivers was seen as potentially useful by the study participants. This would potentially allow less confusion and a higher level of informal care when both professional and nonprofessional caregivers were helping the same individuals. It was suggested that the course content would be adjusted to the user group, but that similar themes or topics of relevance to the organization would be available on the informal caregivers’ own devices, and that new content could be delivered as seen fit.

For other professional caregiving groups such as doctors, nurses, and therapists, it was seen by several study participants as beneficial that these groups “*could see the same material*” as the study participants, primarily to reduce confusion when transferring care duties, as these groups were often seen as having a lower level of knowledge regarding older adult care in their professions. Through the use of the app, these groups could gain and adhere to the same knowledge base. Some study participants expected that these other professions could, for various reasons, not prioritize the same educational initiatives as the older adult care professionals, and thus the digital microlearning app was more likely to be used due to its added advantage regarding time and availability.

Some disadvantages were also noted in using the digital microlearning app regarding meeting future competence development needs. These included some local routines that did not permit the use of personal mobile devices while interacting with care receivers, the inability to install, or overall inaccessibility of organization-provided mobile devices or internet connection. Hygiene aspects related to digital devices were also raised as a concern; “*yet another time I need to think about washing my hands*” was 1 response when deciding whether to take up their mobile to do part of a course. These were seen as general digitalization challenges that some organizations had not yet overcome. One team manager stated regarding this issue:

We say all the time that (the mobile phones) they should not be in our pockets when we’re on the floor, but in our bags instead… but at the same time we need to be more digital. We give very mixed signals to our employees about this.

For those organizations that had come further in their digitalization, the app’s lack of integration with existing digital administrative platforms, or within existing workflows, was also seen by some as an inconvenience to using it. “Not another self-standing application” with its own login requirements, device of installation, and so forth was a comment fielded by a few employees. Some employees with a lower level of Swedish language competency, dyslexia, or other learning difficulties also felt that the textual content itself was “too academic” or advanced, and thus difficult to understand.

Some employees were slightly dismayed at the inability to use the app as a type of knowledge base that could be used more like a guidebook or encyclopedia, for already attained knowledge or to replace existing local knowledge documentation currently in paper form. While the app was not presented as such a solution, some employees felt that it would have been at least as useful in this role as in providing new knowledge content.

The ability to both read and listen to the content was seen as a potential development of the app, which currently only provides content visually (via text, images, and video). Other audio contents such as podcasts within the application were also suggested as complementary material to the course content. Varying the course content for different professional roles using it was also seen as a potentially useful development.

## Discussion

### Principal Results

Our results show that app-based digital microlearning appears to be an appropriate new, person-centered method for providing continuing educational development within the older adult care setting. The proposed advantages of the digital microlearning app were largely confirmed by the participants’ perceptions, particularly regarding ease of use and accessibility, and efficiency and timeliness of knowledge delivery.

While some knowledge areas were deemed appropriate for delivery through the app, others requiring more discussion or reflection were viewed as less appropriate. In these cases, the microlearning method was thought to be more appropriate as a complement or addition to previously used competence development methods. This suggests a combined tailored approach to course theme, and content is advisable when using digital microlearning for complex themes, and that standalone courses conducted via such an app should be appropriately limited in terms of scope, interpretability, and dimensional complexity.

The majority of the participants partially or entirely disagreed that new knowledge had been attained following the dementia course may be explained by the initial knowledge level of the participating organizations. The employees of 1 participating organization appear to have accounted for most of the disagreement, and this organization’s employees had the longest operational careers within older adult care of all participants. Since the themes for the 2 courses conducted within the study were predetermined without direct input from the participating organizations, the material may have been perceived as repetitive or less necessary about current knowledge needs. Similarly, the majority that disagreed that workplace satisfaction had increased following the COVID-19 course were also characterized by longer employment histories. This might suggest that the microlearning courses used in the study provided greater benefits for younger or newer employees in organizations that had more apparent knowledge needs within the course themes. Microlearning courses are, outside of this study, designed and provided in accordance with user organizations’ needs and wishes, which would likely lead to more positive ratings of the app’s perceived usefulness. Our results therefore advise carefully considering organization-specific aspects and context both when implementing and assessing the effects of digital microlearning in health and social care organizations.

Participants perceived an increase in confidence in knowledge and work performance benefits following their use of the app. Ideally, the use of digital microlearning would then lead to better health outcomes and improved safety and quality of life for care receivers. This would create both organizational improvements sooner and increase the return on investment for competency development initiatives. Although this study did not measure such health, safety, and quality of life outcomes among care receivers, the obtained results suggest that such outcomes could logically be achieved. Further research that measures such outcomes would be justified in future studies of digital microlearning.

The app development suggestions from the study participants, including audio-based functions, searchable reference-type functions, and adaptable text content for user groups, suggest an interest in using the capabilities of digital platforms even more than at present. Combined with the overall positive assessment of the app, this can be interpreted as a willingness to digitalize, including the more traditional aspects of health and social care workplaces such as education and training. These results also support upscaled testing within the codevelopment process for the digital microlearning app.

Considering the challenges regarding staff composition and recruitment within the older adult care sector, research regarding competency development via digital methods might help identify ways of increasing the attractiveness of the caregiving professions. The assessed digital microlearning app demonstrated good potential to assist organizational transformation through tailored employee development with little perceived resistance. The benefits of this may be more pronounced among younger or less experienced employees with more diverse backgrounds, which would fit well with currently dominating demographic trends in health and social care personnel management.

### Limitations

The research was conducted in Swedish municipal care settings and used largely user-assessed outcomes. Future research should focus on objective evaluation of health-related outcomes, quality of care, and employee health outcomes following digital microlearning interventions.

### Comparison With Prior Work

Our results are in line with a recent systematic review [6] of 17 studies that demonstrated a positive effect of microlearning on the knowledge and confidence of health profession students in performing procedures, retaining knowledge, studying, and engaging in collaborative learning.

### Conclusions

This study contributes to the currently limited empirical evidence related to digital microlearning [12]. The digital microlearning app demonstrated positive effects at the testing stage in the design thinking model and appears mature for implementation in wider but more tailored use. Competence development strategies should consider digital microlearning as a potential intervention in health and social care organizations.

## Appendix

Multimedia Appendix 1 Course conduct survey statements.

Multimedia Appendix 2 App use survey statements.

Multimedia Appendix 3 Interview guide for the semistructured interviews with employees.
