# Supplementary material for: Digital Microlearning for Training and Competency Development of Older Adult Care Personnel: Mixed Methods Intervention Study to Assess Needs, Effectiveness, and Areas of Application
Source: JMIR Med Educ. 2023 Dec 4;9:e45177. doi: 10.2196/45177 (PMC10728783; doi:10.2196/45177)
Supplement: Multimedia Appendix 1 [file mededu_v9i1e45177_app1.docx]

Complete version of statements in the surveys to which study participants related their experiences of conducting courses. Translated from Swedish. Response alternatives to these statements were: Completely disagree, Mostly disagree, Both agree and disagree, Mostly agree, and Completely agree.

- I will remember the (specific course education) content well.
- I very much enjoyed the (specific course education).
- The (specific course education) was very useful in my work.
- Participation in this kind of education was very beneficial for my work.
- I learned many new things from the (specific course education).
- I often used the knowledge obtained from the (specific course education) in my daily work.
- I successfully applied the content from the (specific course education) in my daily work.
- After the (specific course education) I have become more satisfied with my work.
- My work performance has improved by applying the (specific course education) content.
- Overall, I feel that work processes have improved at my workplace by applying the (specific course education) content.
- Overall, I feel that my work environment has improved by applying the (specific course education) content.
