# Supplementary material for: Digital Microlearning for Training and Competency Development of Older Adult Care Personnel: Mixed Methods Intervention Study to Assess Needs, Effectiveness, and Areas of Application
Source: JMIR Med Educ. 2023 Dec 4;9:e45177. doi: 10.2196/45177 (PMC10728783; doi:10.2196/45177)
Supplement: Multimedia Appendix 2 [file mededu_v9i1e45177_app2.docx]

Complete version of statements in the surveys to which study participants related their experiences while using the application. Translated from Swedish. Response alternatives to these statements were: Completely disagree, Mostly disagree, Both agree and disagree, Mostly agree, and Completely agree.

- I would use the application regularly.
- I found the application easy to use.
- I found the different parts of the application to be well integrated.
- I quickly learned how to use the application.
- I feel comfortable in using the application.
