# Supplementary material for: Digital Microlearning for Training and Competency Development of Older Adult Care Personnel: Mixed Methods Intervention Study to Assess Needs, Effectiveness, and Areas of Application
Source: JMIR Med Educ. 2023 Dec 4;9:e45177. doi: 10.2196/45177 (PMC10728783; doi:10.2196/45177)
Supplement: Multimedia Appendix 3 [file mededu_v9i1e45177_app3.docx]

Interview guide for qualitative interviews with employees. Translated from Swedish. Note that due to the semi-structured format, not all questions may have been posed exactly as written, and additional questions may also have been posed.

Follow-up after conducting the modules

1) What types of follow-up did you do after implementing the modules in the application?

- Was the application discussed with the manager(s)? Other employees?
  - Did you get enough time to discuss or supplement the content of the application after you had completed the module(s)?
- How were any problems or concerns resolved after implementing the modules in the application?
  - How do you think it worked?
  - Was it suitable?
  - Did the follow-up support/help you in your professional practice?

2) What types of follow-up would you like to do if you continue to use the application?

- How would the follow-up work in practical terms, if you could choose?

Possible areas of use

Instruction: In the following questions, imagine the same application that you have used, but that the content of the training modules could be increased or adapted to your wishes or needs.

1) What types of training or development efforts do you think would fit in such an application?

- How do you think it differs from training or development efforts that you have used in the past? (e.g. lecture, internship, study circle)
- How would the location of the training/development effort be affected for you if you used the application, if at all?
- Do you think there would be any difference for you in terms of time to use the application for training/development instead of the methods you have used before?
- What other resources (eg external, internal, people, books) do you think would be needed or affected if using the application instead of the methods you have used before?

2) How do you see the possibility of others (e.g. Colleagues, managers who have not tested the app yet) carrying out training/development efforts in such an application?

- How do you think knowledge acquired from the application would be used in daily work, if at all?
  - How do you think their learning via the application would affect you in your daily work? In other situations?

Comparison with other methods/channels

Instruction: In the following questions, imagine the same application that you have used, but that the content of the training modules could be increased or adapted to your wishes or needs.

1) Which method do you think would best meet the training needs you felt existed among the care staff? The application or one of the previously used methods?

- Why?

2) Which method do you think would best meet the training needs you felt you had as an employee? The application or one of the previously used methods?

- Why?

3) What types of support would you like in an application-based training/development method?

- What would be required to bring these about?
  - How would it feel to fulfill these needs?
  - What would it mean for your professional practice?

4) What advantages and disadvantages do you think there would be in an application-based training/development method during a pandemic-like situation?

- Who would be there if it were normal, i.e. no pandemic?
  - Now that you have been able to try the application, how does it feel in your professional practice compared to before the pandemic?

Rounding off

1) Overall, how suitable do you think the application is as a platform for training/development for you?

- Why?
